# Supplementary material for: MetaRibo-Seq measures translation in microbiomes
Source: Nat Commun. 2020 Jun 29;11:3268. doi: 10.1038/s41467-020-17081-z (PMC7324362; doi:10.1038/s41467-020-17081-z)
Supplement: Supplementary file 10 — Supplementary Data 7 [file 41467_2020_17081_MOESM10_ESM.zip › File2/Confidence_High_Taxonomy/395324_out.krona.html]

Javascript must be enabled to view this page.

members
magnitude
magnitudeUnassigned
count
unassigned
taxon
rank

395324\_out

13

superkingdom
2
13

phylum
13
1239

class
13
186801

13
1

SRS015578\_contig\_number\_5827
order
186802

family
1
31979

1
1649459

SRS893256\_contig\_number\_contig-100\_215.216
genus

186803
family

SRS893259\_contig\_number\_2023
1
11

genus
572511
10


SRS012273\_contig\_number\_36692SRS012902\_contig\_number\_153SRS017521\_contig\_number\_30854SRS075398\_contig\_number\_contig-100\_579.21340SRS097889\_contig\_number\_38553SRS1041095\_contig\_number\_2121SRS1055043\_contig\_number\_23064SRS143780\_contig\_number\_25702SRS148511\_contig\_number\_17334
species
9
33038

1
33039
species

SRS057717\_contig\_number\_6719
